# Comparative assessment of flexural strength of monolithic zirconia with different thicknesses and two sintering techniques

**DOI:** 10.1002/cre2.856

**Published:** 2024-05-31

**Authors:** Nilofar Karbasian, Amirhossein Fathi, Pirooz Givehchian, Saeed Nosouhian, Mohammad Jamshidian, Farhad Almassi, Ali Fazeli

**Affiliations:** ^1^ Dental Students’ Research Committee, School of Dentistry Isfahan University of Medical Sciences Isfahan Iran; ^2^ Department of Prosthodontics, School of Dentistry, Dental Materials Research Center Isfahan University of Medical Sciences Isfahan Iran; ^3^ Department of Prosthodontics, School of Dentistry, Dental Implants Research Center Isfahan University of Medical Sciences Isfahan Iran; ^4^ Dental Prosthesis Student, Faculty of Dentistry Isfahan University of Medical Sciences Isfahan Iran

**Keywords:** flexural strength, sintering, zirconium oxide

## Abstract

**Objectives:**

This study aimed to compare the flexural strength of monolithic zirconia with different thicknesses and two sintering techniques.

**Materials and Methods:**

This in vitro, experimental study was conducted on 28 monolithic zirconia discs with 10 mm diameter and 0.5 (*n* = 14) and 1.2 mm (*n* = 14) thickness. Each group was divided into two subgroups (*n* = 7) for fast (60 min) and conventional (120 min) sintering at 1450°C. After sintering, the specimens were thermocycled and their flexural strength was measured by piston‐on‐3‐balls technique in a universal testing machine (0.5 mm/min, 1.2 mm pin diameter). Data were analyzed by the Weibull test, one‐way analysis of variance, and Tukey's test (*α* = .05).

**Results:**

The flexural strength of specimens with 1.2 mm thickness was significantly higher than that of specimens with 0.5 mm thickness (*p* < .05). The flexural strength of 1.2 mm/120‐min group was slightly, but not significantly, higher than that of 1.2 mm/60‐min group (*p* > .05). The flexural strength of 0.5 mm/120‐min group was slightly, but not significantly, higher than that of 0.5 mm/60‐min group (*p* > .05).

**Conclusion:**

The increase in thickness of monolithic zirconia increases its flexural strength; however, increasing the sintering time appears to have no significant effect on the flexural strength of monolithic zirconia.

## 1 INTRODUCTION

The increasing demand for all‐ceramic restorations due to their excellent esthetics, optimal biocompatibility, and acceptable strength led to the introduction of different ceramics with more favorable properties such as monolithic zirconia (Ebeid et al., 2014; Ozer et al., 2018; Sulaiman et al., 2015). However, the fragility and low tensile strength of ceramics can compromise their long‐term clinical success. Thus, research is ongoing to improve the strength of ceramics (Denry & Kelly, 2014).

Yttria‐stabilized tetragonal zirconia polycrystal is currently used for implant abutment, framework of fixed partial dentures, and framework of screw‐retained implant restorations due to its excellent esthetics, optimal biocompatibility, lower plaque accumulation, minimal electrical conductivity, and acceptable hardness and fracture resistance (Flinn et al., 2017; Guazzato et al., 2004).

Zirconia restorations can be fabricated by computer‐aided design/computer‐aided manufacturing (CAD/CAM) technology (Coli & Karlsson, 2004). The recommended thickness for ceramic‐veneered crowns is 1.5 mm for the occlusal surface and 1 mm for the cervical surface (Edelhoff & Sorensen, 2002). However, the manufacturers claim that zirconia can be used with lower thicknesses as well (Beuer et al., 2012). Non‐veneered zirconia currently has higher popularity since it does not have the problems related to cracking of the veneering, and is also suitable for use in patients with parafunctional habits (Traini et al., 2015). A unique property of yttria‐stabilized tetragonal zirconia polycrystal is that it prevents crack propagation when sintered (Hannink et al., 2000; Kohorst et al., 2011).

Pure zirconia has three different crystalline phases at different atmospheric temperatures: (I) the monolithic phase at temperatures below 1170°C, (II) the tetragonal phase at temperatures between 1170°C and 2370°C, and (III) the cubic phase at temperatures above 2370°C to the melting point (Chevalier, 2006). To stabilize the zirconia in the tetragonal phase at room temperature, some stabilizing oxides such as calcium oxide, magnesium oxide, selenium oxide, and yttrium oxide are added to pure zirconia (Denry & Kelly, 2008).

Tensile stresses at the tip of a crack cause tetragonal to monoclinic phase transformation, which results in 3%–5% volumetric expansion. This increase in volume causes compressive stress at the crack tip, which resists external tensile stresses, and prevents or decelerates crack propagation (Bachhav & Aras, 2011). Tetragonal to monoclinic phase transformation may be induced by hydrothermal aging, acidification when eating, masticatory cycles, and surface wear, which is referred to as low‐temperature degradation. Thus, phase transformation gradually progresses from the surface to the depth of the material (Ebeid et al., 2018).

The zirconia sintering process includes heating, baking, and cooling (Marinis et al., 2013). Although the use of CAD/CAM technology significantly decreases the clinical working time, zirconia sintering takes a couple of hours (Miyazaki et al., 2013). Also, 20%–30% shrinkage occurs in the process of sintering (Manicone et al., 2007). Zirconia sintering at very high temperatures decreases its flexural strength and leads to the migration of yttria particles. Different techniques of zirconia sintering can significantly affect its microstructure and properties. It appears that differences in sintered holding time in the process of sintering can affect the size of particles, their microscopic structure, translucency, and strength of zirconia. However, controversy exists regarding the effect of sintering time and temperature on translucency, microscopic structure, and strength of zirconia ceramic core (Stawarczyk et al., 2013).

Monolithic zirconia restorations do not undergo chipping and have acceptable mechanical strength, lower thickness, optimal esthetics, faster production, and lower cost (Sen et al., 2018).

Hydrothermal aging of zirconia, which is also known as low‐temperature degradation, can occur at temperatures between 65°C and 500°C in water or other liquids (Siarampi et al., 2014). Although this mechanism occurs very slowly in the oral cavity, zirconia restorations may undergo aging and experience a reduction in strength due to constant moisture, thermal and pH alterations, and frequent occlusal loads as well as parafunctional habits (Lughi & Clarke, 2005). Factors such as chemical composition, the brand of zirconia, the thickness of restoration, and fabrication method can also affect the resistance of zirconia restorations to aging (Khayat et al., 2018).

Considering all the above, this study aimed to compare the flexural strength of monolithic zirconia in two different thicknesses and two sintering techniques. The first null hypothesis was that no significant difference would be found between the two thicknesses of monolithic zirconia regarding flexural strength. The second null hypothesis was that no significant difference would be found in the flexural strength of monolithic zirconia restorations fabricated by the two sintering techniques.

## 2 MATERIALS AND METHODS

In this in vitro, experimental study, the sample size was calculated to be 7 in each of the four groups assuming *α* = .05, *β* = .2, study power of 80%, and minimum significant difference (*d*) of 307 MPa in the mean flexural strength between the two thicknesses.

### 2.1 Specimen preparation

A total of 28 monolithic zirconia discs were designed with the desired dimensions and thicknesses using SolidWorks 2019 software and milled by a CAD/CAM system (IMES‐ICORE Coritec 350i) using a high‐translucent monolithic zirconia block (Vivid TransZir ML, Zolid fx UT translucency 49%) by taking into account 1.246% volumetric shrinkage as stated by the manufacturer. All discs had 10 mm diameter. Of 28 discs, 14 had 1.2 mm thickness and 14 had 0.5 mm thickness. The discs in each group were further divided into two subgroups (*n* = 7) for fast (60 min) and conventional (120 min) sintering at 1450°C.

### 2.2 Sintering

Before sintering, the surface of the discs was polished with diamond discs and silicon carbide abrasive paper to reach the required thickness. All zirconia discs were then immersed in an ultrasonic bath (Ultrasonic S30, Sky dental supply) for 15 min at 30°C and dried at room temperature. Sintering of the discs was performed in a sintering furnace (VITA ZYRCOMAT 6000 MS, Vita Zahnfabrik).

One subgroup of 1.2 mm discs (*n* = 7) and one subgroup of 0.5 mm discs (*n* = 7) were conventionally sintered at 1450°C with 120 min of sinter‐holding time. The rate of temperature rise was 17°C/min until reaching 1450°C. Next, the discs were sintered at this temperature for 120 min and were then gradually cooled.

The remaining two subgroups were fast‐sintered at 1450°C with a sinter‐holding time of 60 min. The rate of temperature rise was 17°C/min until reaching 1450°C. Next, the discs were sintered at this temperature for 60 min and were then gradually cooled.

### 2.3 Aging

After sintering, all discs underwent aging in a thermocycler (Delta Tpo 2) for 3500 cycles between 5°C and 55°C with a dwell time of 60 s at 5°C, a transfer time of 10 s, a dwell time of 20 s at 55°C, and a subsequent transfer time of 10 s to simulate the clinical setting (Alqahtani & Almansour, 2018).

### 2.4 Flexural strength test

All discs were transferred to a universal testing machine (Electromechanical Universal Testing, Walter+Bai) and subjected to a piston‐on‐3‐balls test following ISO 6872:2015 protocol. In this test, three steel balls, each with a 3.2 mm diameter, are located in a circular plane with a 6 mm diameter and 120° angle. The load was applied by a steel cylinder with a 1.25 mm diameter to the center of each disc. The discs were placed on the balls such that the load was applied right to their center. As soon as the cylinder tip contacted the disc surface, compressive load application started at a crosshead speed of 0.5 mm/min and gradually increased until disc fracture. The load at fracture was recorded in Newtons (N). The flexural strength was calculated using the formula below:

$$\sigma =-0/238\;7P\left(X‐Y\right)/d^{2},$$

where *σ* is the flexural strength in megapascals (MPa), *P* is the fracture load in Newtons (N), *d* is the disc thickness in millimeters (mm), and *Y* and *X* were calculated as follows:

$$X=\left(1+ט\right)\ln\;{\left(r_{2}/r_{3}\right)}^{2}+[(1-ט)/2]{\left(r_{2}/r_{3}\right)}^{2},$$

$$Y=\left(1+ט\right)\left[1+\;\ln\;{\left(r_{1}/r_{3}\right)}^{2}\right]+\left(1-ט\right){\left(r_{1}/r_{3}\right)}^{2},$$

where ט is the Poisson's ratio (0.25), *r*_1_ is the radius of the holding plane, *r*_2_ is the radius of the area under load application, and *r*_3_ is the disc radius (Ebeid et al., 2018).

### 2.5 Statistical analysis

Data were analyzed using SPSS version 21. The normal distribution of data was evaluated by the Weibull test using the respective software (Weibull‐Ease 16.0; Applications Research Inc.). The Weibull modulus (*m*) was calculated using the formula below through the gradient of the line between ln{ln(1/1‐*P*_f_)} and ln(*σ*):

$$P_{f}\left(\sigma_{i}\right)=\left(i-0/5\right)/N,$$

where *P*_f_(*σ*_i_) is the risk of fracture, *i* is the specimen number, and *N* is the total number of specimens. The effect of zirconia disc thickness and sintering technique on flexural strength was analyzed by one‐way analysis of variance (ANOVA). Pairwise comparisons were carried out by Tukey's test with a 95% confidence interval (CI). *p* < .05 was considered statistically significant.

## 3 RESULTS

Table 1 presents the measures of central dispersion for the flexural strength of the four groups. One‐way ANOVA showed a significant difference in flexural strength among the four groups (*p* < .001). Pairwise comparisons by the Tukey's test (Table 2) showed that the flexural strength of specimens in the 0.5 mm thickness/120‐min group was lower than that of 1.2 mm/60‐min (*p* = .001) and 1.2 mm/120 min groups (*p* = .016). Also, the flexural strength of 0.5 mm/60‐min group was significantly lower than that of 1.2 mm/60‐min (*p* = .001) and 1.2 mm/120‐min (*p* = .016) group. No other significant differences were noted (*p* > .05).

**Table 1:** Measures of central dispersion for flexural strength (MPa) of the four groups (*n* = 7).

Group	Mean	Standard deviation	95% confidence interval for mean		Minimum	Maximum
			Lower bound	Upper bound		
0.5 mm and 120 min	367.6945	55.95550	315.9443	419.4447	274.09	428.88
0.5 mm and 60 min	367.5764	38.25780	332.1938	402.9589	324.64	440.06
1.2 mm and 120 min	510.2010	46.59102	467.1115	553.2905	435.81	557.23
1.2 mm and 60 min	467.5458	79.61427	393.9149	541.1767	351.12	577.30
Total	428.2544	83.41810	395.9083	460.6006	274.09	577.30

**Table 2:** Pairwise comparisons of the groups regarding flexural strength.

Group (I)	Group (J)	Mean difference (I‐J)	Standard error	*p*‐Value	95% confidence interval	
					Lower bound	Upper bound
0.5 mm and 120 min	0.5 mm and 60 min	−0.11816	30.59392	1.000	−84.5148	84.2785
	1.2 mm and 60 min	−142.62462	30.59392	0.001	−227.0213	−58.2280
	1.2 mm and 120 min	−99.96941	30.59392	0.016	−184.3661	−15.5727
0.5 mm and 60 min	1.2 mm and 60 min	−142.50646	30.59392	0.001	−226.9031	−58.1098
	1.2 mm and 120 min	−99.85125	30.59392	0.016	−184.2479	−15.4546
1.2 mm and 120 min	1.2 mm and 60 min	−42.65522	30.59392	0.515	−127.0519	41.7414

**Figure 1:**
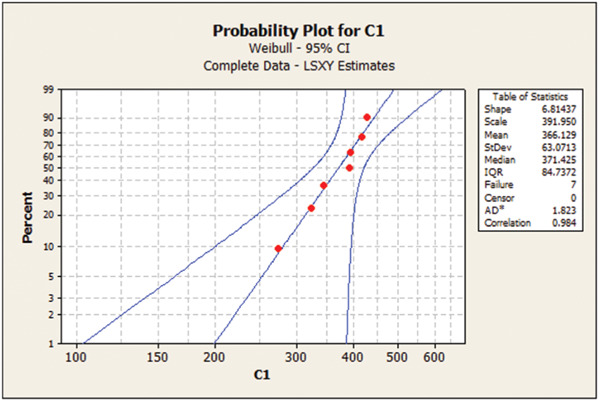
Group 1 (0.5 mm/120 min).

**Figure 2:**
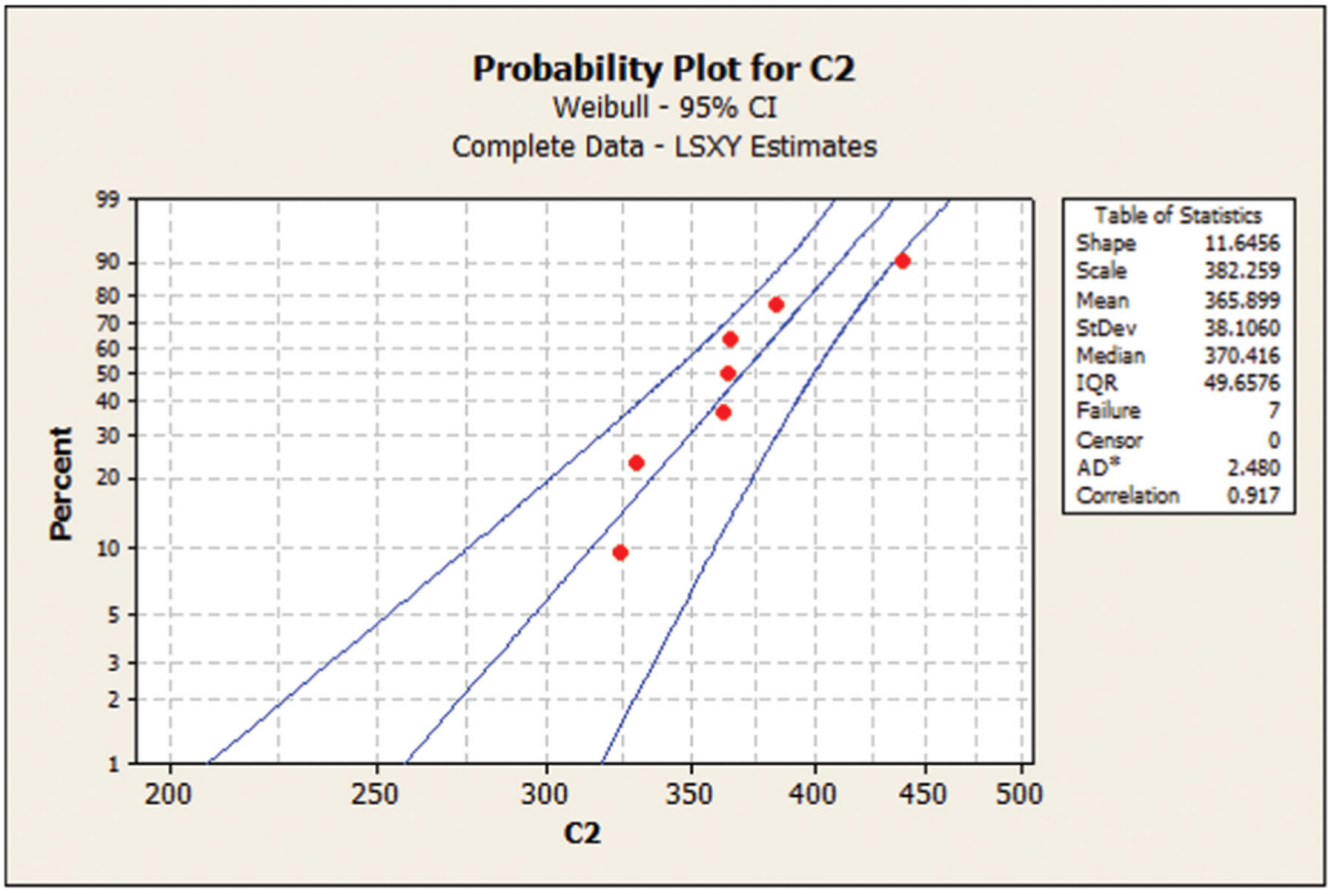
Group 2 (0.5 mm/60‐min).

**Figure 3:**
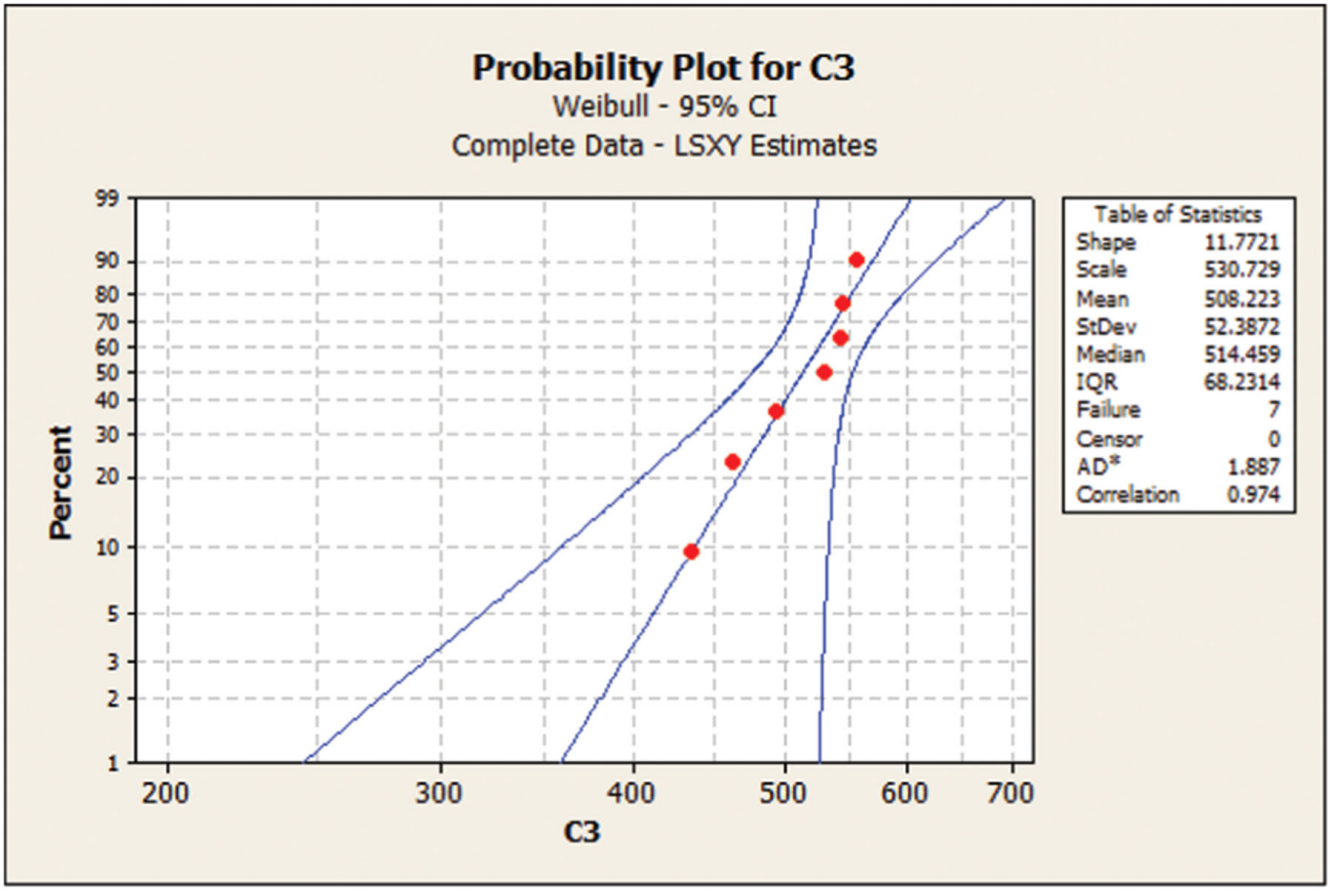
Group 3 (1.2 mm/120 min).

**Figure 4:**
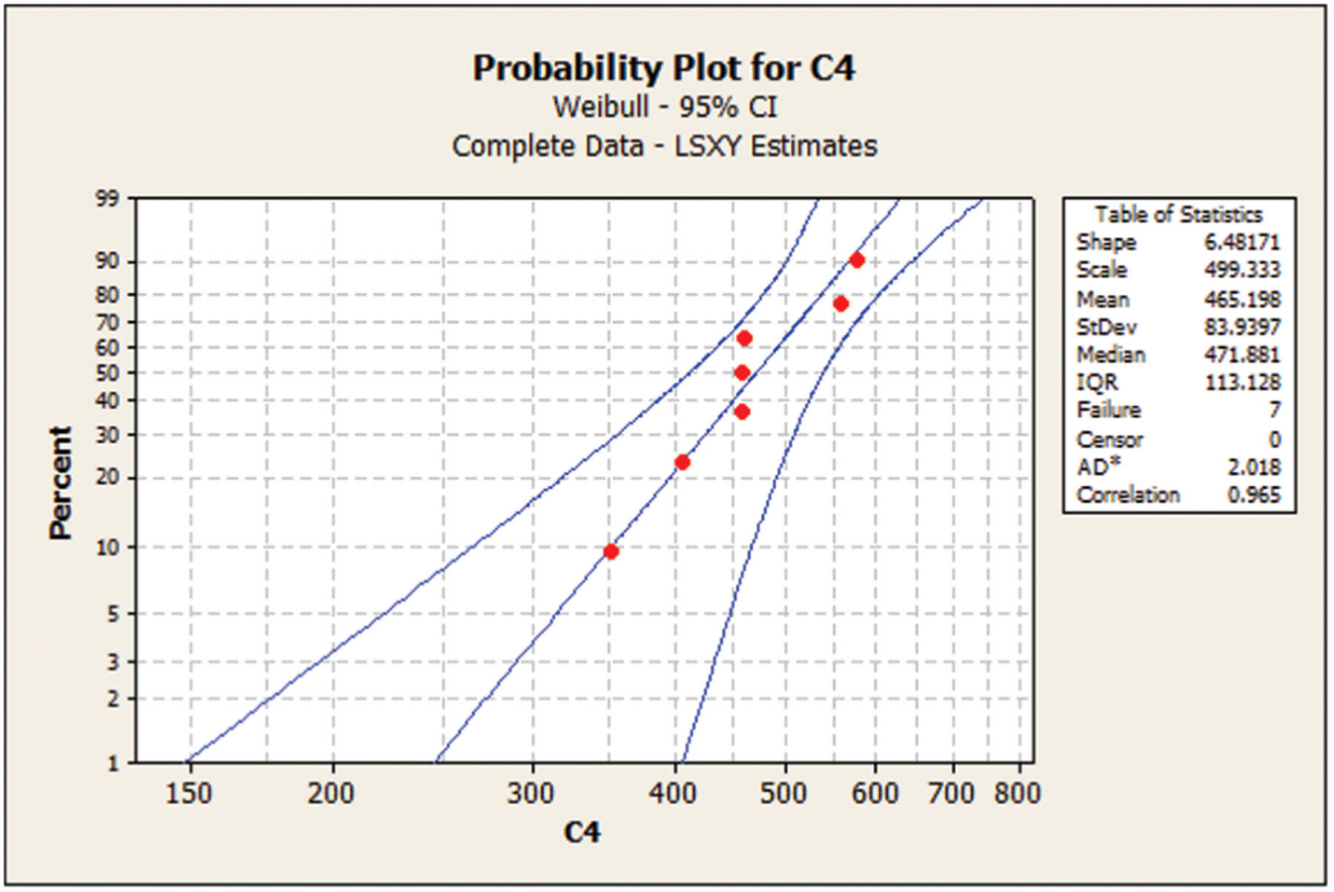
Group 4 (1.2 mm/60‐min).

Table 3 presents the Weibull calculations and the m parameter. As shown, the m value was the highest for 1.2 mm/120‐min group, indicating low deviation and dispersion in mechanical properties in this group. Figures 1, 2, 3, 4 show the Weibull probability plots for the four groups.

**Table 3:** Weibull calculations and m parameter.

*i*	Group	Thickness	Time	Strength *σ* (MPa)	*P*_f_	Ln[Ln[1/1‐P_f_]]	Ln(σ)
1.00	4	1.20	60.00	545.30	545.30	0.03	−3.35
2.00	4	1.20	60.00	557.23	557.23	0.07	−2.64
3.00	4	1.20	60.00	495.13	495.13	0.10	−2.21
4.00	4	1.20	60.00	531.53	531.53	0.14	−1.91
5.00	4	1.20	60.00	435.81	435.81	0.17	−1.66
6.00	4	1.20	60.00	462.79	462.79	0.21	−1.46
7.00	4	1.20	60.00	543.63	543.63	0.24	−1.29
8.00	2	0.50	60.00	416.73	416.73	0.28	−1.13
9.00	2	0.50	60.00	392.94	392.94	0.31	−0.99
10.00	2	0.50	60.00	274.09	274.09	0.34	−0.86
11.00	2	0.50	60.00	344.07	344.07	0.38	−0.74
12.00	2	0.50	60.00	394.04	394.04	0.41	−0.63
13.00	2	0.50	60.00	323.11	323.11	0.45	−0.52
14.00	2	0.50	60.00	428.88	428.88	0.48	−0.42
15.00	3	1.20	120.00	458.67	458.67	0.52	−0.32
16.00	3	1.20	120.00	406.77	406.77	0.55	−0.22
17.00	3	1.20	120.00	559.90	559.90	0.59	−0.13
18.00	3	1.20	120.00	459.95	459.95	0.62	−0.03
19.00	3	1.20	120.00	459.10	459.10	0.66	0.06
20.00	3	1.20	120.00	351.12	351.12	0.69	0.16
21.00	3	1.20	120.00	577.30	577.30	0.72	0.25
22.00	1	0.50	120.00	440.06	440.06	0.76	0.35
23.00	1	0.50	120.00	364.85	364.85	0.79	0.45
24.00	1	0.50	120.00	330.63	330.63	0.83	0.56
25.00	1	0.50	120.00	324.64	324.64	0.86	0.68
26.00	1	0.50	120.00	365.95	365.95	0.90	0.82
27.00	1	0.50	120.00	384.41	384.41	0.93	0.98
28.00	1	0.50	120.00	362.48	362.48	0.97	1.21

*Note*: 1: 0.5 mm/120 min; 2: 0.5 mm/60‐min; 3: 1.2 mm/120 min; 4: 1.2 mm/60 min.

## 4 DISCUSSION

This study compared the flexural strength of monolithic zirconia in two different thicknesses and two sintering techniques. The first null hypothesis was that no significant difference would be found between the two thicknesses of monolithic zirconia regarding flexural strength. The second null hypothesis was that no significant difference would be found in the flexural strength of monolithic zirconia restorations fabricated by the two sintering techniques. The results showed that the flexural strength of specimens with 1.2 mm thickness was significantly higher than that of specimens with 0.5 mm thickness (*p* < .05). Thus, the first null hypothesis of the study was rejected. The flexural strength of 1.2 mm/120‐min group was slightly, but not significantly, higher than that of 1.2 mm/60‐min group (*p* > .05). The flexural strength of 0.5 mm/120‐min group was slightly, but not significantly, higher than that of 0.5 mm/60‐min group (*p* > .05). Thus, the second null hypothesis of the study was accepted.

Although the appropriate thickness for a monolithic zirconia crown is 1–1.5 mm at the occlusal surface (Ozer et al., 2018), 1.2‐ and 0.5‐mm thicknesses were evaluated in this study to find the minimum thickness with acceptable flexural strength. Slightly higher flexural strength in the group with longer sintering time may be due to the maturation of crystalline structures, fewer defects in marginal particles, and an increase in particle size (Jiang et al., 2011; Stawarczyk et al., 2013). The sintering process eliminates the gaps between the particles in granules. As the sintering time increases, zirconia particles acquire a greater capacity to attach to each other. Also, gaps between the marginal granules decrease, material density increases and zirconia strength is reinforced (Chen & Wang, 2000; Stawarczyk et al., 2013). Matsuzaki et al. (2015) reported that the flexural strength of monolithic zirconia with 1 mm thickness was higher than that of lithium disilicate specimens with 1.5 mm thickness, and alumina ceramic specimens with 2–2.9 mm thickness. They concluded that zirconia can be used with 1 mm thickness (Matsuzaki et al., 2015). Sun et al. (2014) reported that increasing the thickness of monolithic zirconia from 0.6 mm to 1.5 mm significantly increased its flexural strength; nonetheless, 0.6 mm thickness provided adequate flexural strength for posterior restorations. Ozer et al. (2018) assessed the effect of monolithic zirconia thickness on flexural strength and showed that the flexural strength of 1.3 mm zirconia was higher than that of 0.8 mm thickness. Juntavee and Attashu (2018) evaluated the effect of sintering on the flexural strength of monolithic zirconia and showed that sintering at higher temperatures and for a longer period of time yielded higher flexural strength than shorter sintering time at a lower temperature.

The Weibull modulus is a statistical measure used to assess the reliability and consistency of material strength by analyzing the distribution of defects within a material. It is used to evaluate the reliability of flexural strength values obtained from different groups of zirconia samples. A higher Weibull modulus suggests a lower dispersion of data points, indicating a more uniform distribution of defects within the material (Wang & Shao, 2014). This uniformity implies greater homogeneity in mechanical properties across the material, leading to higher reliability in the obtained flexural strength values. Essentially, a higher Weibull modulus signifies a more consistent and predictable behavior of the material in terms of strength. In this study, the group with a Weibull modulus of 1.2 mm/120 min showed a higher value compared to other groups. This higher modulus indicates that this group had lower dispersion in flexural strength values, signifying a more uniform distribution of defects within the material and, consequently, higher reliability in the obtained flexural strength values for this group.

Future studies with a larger sample size are required on other thicknesses and other sintering temperatures of zirconia.

## 5 CONCLUSION

The increase in thickness of monolithic zirconia increases its flexural strength; however, increasing the sintering time appears to have no significant effect on the flexural strength of monolithic zirconia.

## AUTHOR CONTRIBUTIOS

**Nilofar Karbasian**: Acquisition of data; analysis of data; drafting of article and/or critical revision; final approval of manuscript. **Amirhossein Fathi**: Conception and design of the study; final approval of the manuscript; analysis of data. **Pirooz Givehchian**: Conception and design of the study; acquisition of data; drafting of article and/or critical revision; final approval of the manuscript. **Saeed Nosouhian**: Conception and design of study; analysis of data; drafting of article and/or critical revision; final approval of manuscript. **Mohammad Jamshidian**: Conception and design of study; acquisition of data; drafting of article and/or critical revision; final approval of manuscript. **Farhad Almassi and Ali Fazeli**: data gathering (make species and run the tests); analysis of data.

## CONFLICT OF INTEREST STATEMENT

The authors declare no conflict of interest.

## Data Availability

The data sets analyzed during the current study are not publicly available due to not having consent from all patients to publicly publish this data but are available from the corresponding author upon reasonable request.
